# Clinical and Molecular Characterization and Discovery of Novel Genetic Mutations of Chinese Patients with *COL2A1*-related Dysplasia

**DOI:** 10.7150/ijbs.38811

**Published:** 2020-01-16

**Authors:** Yang Xu, Li Li, Chun Wang, Hua Yue, Hao Zhang, Jiemei Gu, Weiwei Hu, Lianyong Liu, Zhenlin Zhang

**Affiliations:** 1 Department of Osteoporosis and Bone Diseases, Metabolic Bone Disease and Genetics Research Unit, Shanghai Jiao Tong University Affiliated Sixth People's Hospital, 600 Yishan Road, Shanghai 200233, China; 2 Department of Endocrinology, Punan Hospital of Pudong New District, 279 Linyi Road, Shanghai 200125, China

**Keywords:** * COL2A1-related dysplasia*, * COL2A1*, mutations, phenotype-genotype relation

## Abstract

*COL2A1*-related disorders represent a heterogeneous group of skeletal dysplasias with a wide phenotypic spectrum. Our aim is to characterize the clinical and molecular phenotypes of Chinese patients with *COL2A1*-related dysplasia and to explore their phenotype-genotype relations. Clinical data were collected, physical examinations were conducted, and X-ray radiography and genetic analyses were performed in ten families involving 29 patients with* COL2A1*-related dysplasia. Nine mutations were identified in *COL2A1*, including five novel (c.816+6C>T, p.Gly246Arg, p.Gly678Glu, p.Gly1014Val and p.Ter1488Gln) and four reported previously (p.Gly204Val, p.Arg275Cys, p.Gly504Ser and p.Arg719Cys). Based on clinical features and molecular mutations, the ten families were classified into five definite *COL2A1*-related disorders: four families with spondyloepiphyseal dysplasia congenita (SEDC), three with osteoarthritis with mild chondrodysplasia (OSCPD), one with Czech dysplasia, one with Kniest dysplasia, and one with epiphyseal dysplasia, multiple, with myopia and deafness (EDMMD). Based on genetic testing results, prenatal diagnosis and genetic counseling were accomplished for one female proband with OSCDP. Chinese patients with OSCDP, Czech dysplasia and EDMMD caused by *COL2A1* mutations were first reported, expanding the spectrum of *COL2A1* mutations and the phenotype of *COL2A1*-related disorders and providing further evidence for the phenotype-genotype relations, which may help improve procreative management of *COL2A1*-related disorders.

## Introduction

Type Ⅱ procollagen alpha-1 chain encoded by the *COL2A1* gene is a major component of type Ⅱ collagen expressed in chondrocytes and vitreous humor 1. Mutations in *COL2A1* gene affect endochondral ossification and linear bone growth with structurally abnormal type Ⅱ collagen 2. *COL2A1*-related disorders mostly caused by *COL2A1* mutations represent a heterogeneous group of skeletal dysplasia with a wide phenotypic spectrum, ranging from perinatal death to relatively mild osteoarthritis 3, 4. The unifying manifestations of these disorders are mostly involved in joints and spine, whereas the specific signs and symptoms and severity may vary in the clinic, with extraskeletal (ocular and otolaryngological) abnormalities contributing to phenotypic diversity 5-7. According to the Nosology and Classification of Genetic Skeletal Disorders (2015 revision) 8, *COL2A1*-related dysplasia mainly include achondrogenesis type 2 (ACG2; OMIM# 200610), hypochondrogenesis (HCG; OMIM# 200610), platyspondylic dysplasia, Torrance type (PLSDT; OMIM# 151210), spondyloepiphyseal dysplasia congenita (SEDC; OMIM# 183900), spondyloepiphyseal dysplasia, Strudwick type (SED Strudwick type; OMIM# 184250), Kniest dysplasia (OMIM# 156550), spondylopepripherad dysplasia (SPPD; OMIM# 271700), epiphyseal dysplasia, multiple, with myopia and deafness (EDMMD; OMIM# 132450), osteoarthritis with mild chondrodysplasia (OSCDP; OMIM# 604864), Czech dysplasia (OMIM# 609162), Stickler syndrome type 1 (STL1; OMIM# 108300), and Legg-Calve-Perthes disease (LCPD; OMIM# 150600).

Among the disease-causing mutations in *COL2A1* leading to *COL2A1*-related dysplasia, the majority are missense mutations and the most common type is the substitution in glycine residues. Glycine to serine substitutions cause variable phenotypes (severe and mild) by resulting in alternating zones, while glycine to non-serine substitutions produce more severe phenotypes including HCG or SEDC with severe coxa vara 5. In the non-glycine missense mutations, arginine to cysteine substitution predominates and creates moderate phenotypes with either normal or short stature 9. In-frame deletions and duplications have mainly been detected as the cause of STL1, PLSDT, or Kniest dysplasia, and frameshift or nonsense mutations usually result in normal stature and precocious osteoarthritis 5, 10-12. Splice-site mutations are identified to cause exon skipping in an otospondylomegaepiphyseal dysplasia case 13. In addition to mutation types, the locations of mutations are also correlated to the disease phenotype. For instance, missense mutations in the triple-helical region mostly cause ACG2, HCG and SEDC, while truncation or splice-site mutations in triple-helical or N-propeptide region usually cause STL1 or Kniest dysplasia with extraskeletal manifestations 5. Mutations located in C-propeptide region produce atypical skeletal phenotypes with ocular involvement and mild to moderate growth failure 5.

We have previously reported four Chinese families with SEDC and one family with SPPD, discovering four novel mutations in *COL2A1* including three missense and one frameshift 14, 15. *COL2A1*-related dysplasia greatly reduces the quality of life of the patients and their families, causing huge economic and psychological burden on them 16, 17. Although a large number of *COL2A1* mutations have been reported and the phenotype-genotype relations in *COL2A1*-related dysplasia have been partially elucidated, the clinical and molecular characteristics in Chinese patients with *COL2A1*-related dysplasia remain unclear. The present study revealed several novel mutations in the *COL2A1* gene and their relation to clinical phenotypes was explored. We believe the results from the present study would help improve our understanding of *COL2A1*-associated dysplasia, especially in Chinese population.

## Materials and Methods

### Patients

The present study was reviewed and approved by the Ethics Committee of the Shanghai Jiao Tong University Affiliated Sixth People's Hospital, and written informed consents were obtained from all the adult participants and the children's legal guardians. The study was conducted in accordance with the Declaration of Helsinki. Ten non-consanguineous Chinese families with *COL2A1*-related dysplasia were diagnosed from 2012 to 2019, with 29 affected individuals being tested for clinical and molecular phenotypes. Clinical data, including family history, onset age, height, and skeletal, ocular, and otolaryngological manifestations were attained from the medical records and questionnaires. Radiographs of spines, pelvises, and affected joints were taken and evaluated by an experienced radiologist in blinded fashion. Additionally, 24 unaffected family members among the ten families and 250 healthy volunteers were also recruited as controls in the present study.

### Mutation Analysis

**Figure 1:**
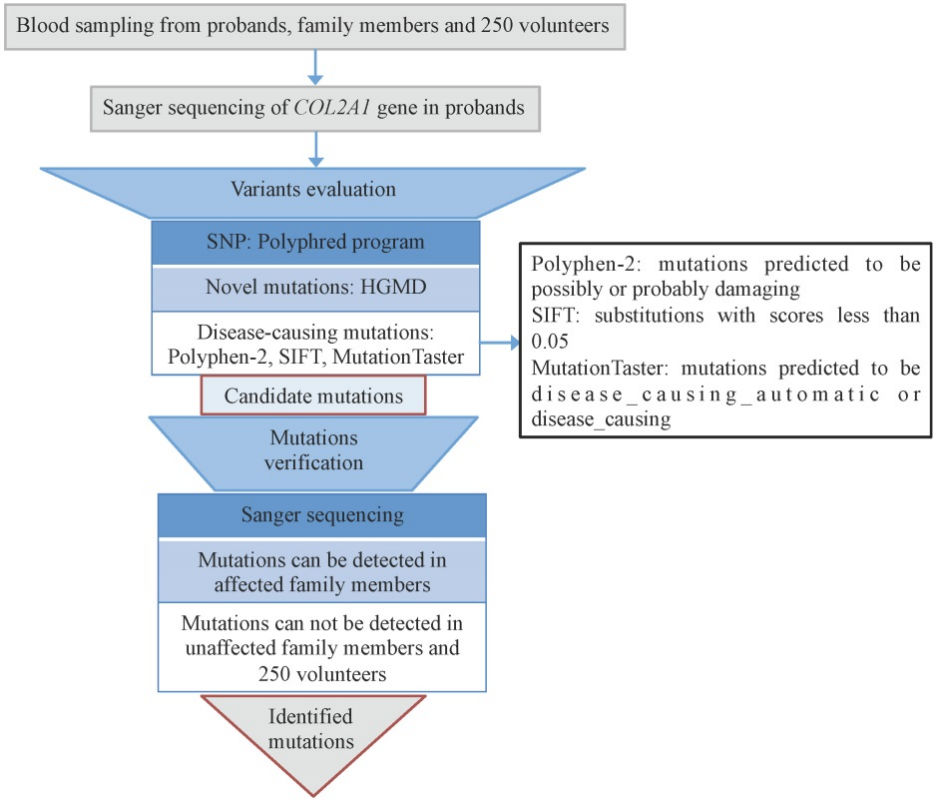
Flowchart of genetic analysis. All the mutations were evaluated through bioinformatics and verified among all the family members and 250 volunteers.

Informed consents were obtained from the ten families and 250 healthy volunteers before blood sampling and DNA analysis. We used QuickGene DNA whole blood kit (Kurabo Industries Ltd., Osaka, Japan) and a Nucleic Acid Isolation system (QuickGene-610L; Autogen, Inc., Holliston, MA, USA) to extract genomic DNA from 2-mL peripheral blood samples of the probands. Direct Sanger sequencing of *COL2A1* gene was accomplished. The full sequence of *COL2A1* was attained from online database (GenBank accession no. NC_000012). All 54 exons and the exon-intron boundaries of *COL2A1* were amplified through PCR using primers designed by the Primer 3 software (http://frodo.wi.mit.edu/cgi-bin/primer3/primer3_www.cgi). The sequencing was performed on PCR products with the BigDye Terminator Cycle Sequencing Ready Reaction Kit (version 3.1; Applied Biosystems; Thermo Fisher Scientific, Inc., Waltham, MA, USA), and the results were analyzed with an ABI Prism 3130 automated sequencer (Thermo Fisher Scientific, Inc.). Single nucleotide polymorphisms (SNPs) were checked by the Polyphred program (http://droog.mbt.wasgington.edu/poly-get.html). Novel mutations were recognized by using HGMD (http://www.hgmd.cf.ac.uk/ac/index.php). Disease-causing mutations were predicted by using Polyphen-2 (http://provean.jcvi.org), SIFT (http://sift.jcvi.org), and MutationTaster (http://www.mutationtaster.org) programs. All the identified mutation sites were verified among the family members and 250 healthy volunteers (Fig. 1).

## Results

### Clinical features

#### Spondyloepiphyseal dysplasia congenita (SEDC)

**Figure 2:**
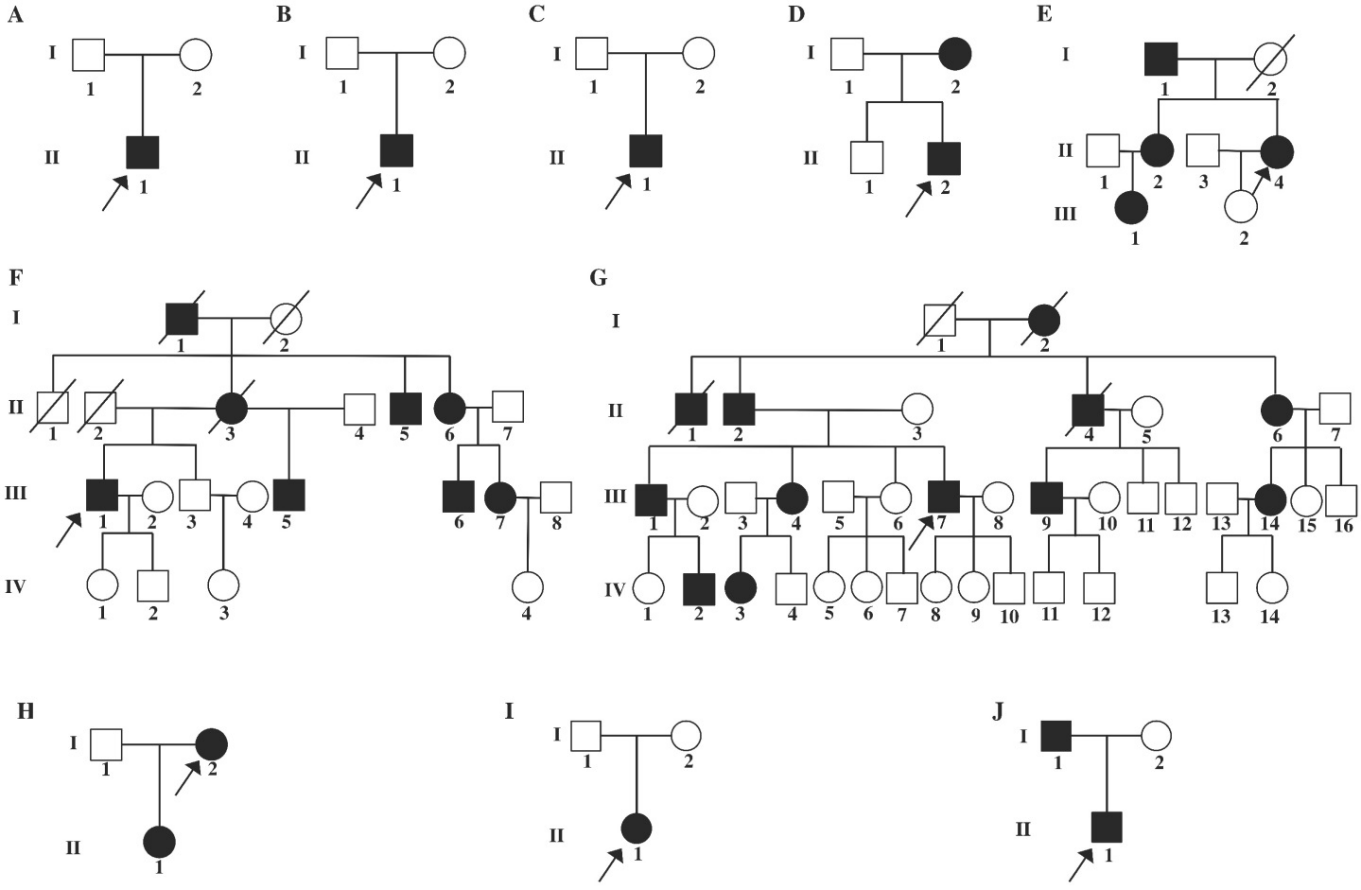
Pedigrees of ten families with COL2A1-related disorders. A: Family 1, B: Family 2, C: Family 3, D: Family 4, E: Family 5; F: Family 6; G: Family 7, H: Family 8, I: Family 9, J: Family 10. Black symbols represent the unaffected individuals. Circles and squares indicate females and males, respectively. Arrows identify the proband in the families. Slashes indicate deceased individuals.

**Figure 3:**
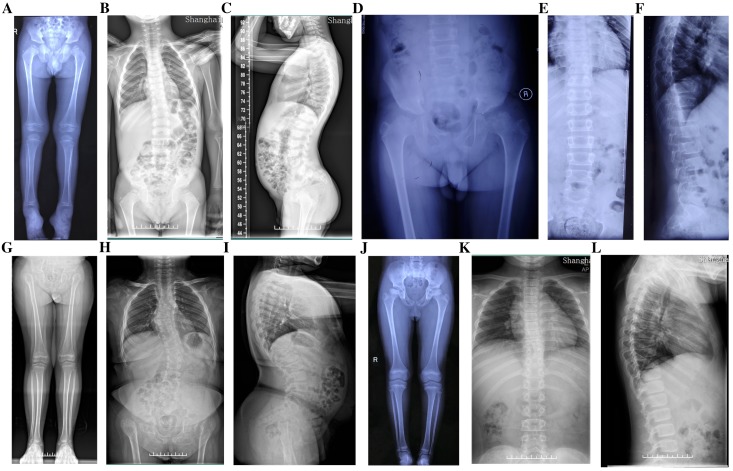
Radiographs of the probands with SEDC. A-C: Full-length X-rays of both lower extremities (A) and spine radiographs (B, C) of the proband in Family 1. Flattening of the acetabular roof, small capital femoral epiphyses, enlarged distal femur metaphyses and ovoid vertebral bodies were displayed. D-F: X-rays of pelvis (D) and spine (E, F) of the proband in Family 2. Partial collapse and uneven density of bilateral femoral heads, flattening of the acetabular roof, shortening of the femoral neck and platyspondyly were shown. G-I: Full-length X-rays of both lower extremities (G) and spine radiographs (H, I) of the proband in Family 3. Small iliac bones, horizontal acetabular roof, coxa vara, very short femoral necks, small and fragmented ossification of capital femoral epiphyses, enlarged distal femur metaphyses, severe scoliosis and moderate platyspondyly were demonstrated. J-L: Full-length X-rays of both lower extremities (J) and spine radiographs (K, L) of the proband in Family 4. Flattening of the acetabular roof, compressed femoral head, shortening of the femoral neck and base of the ilium, distinctly flared metaphyses of femurs and slight platyspondyly were shown.

Four probands from four non-consanguineous families had same initial symptoms of groin pain, limitation in hip mobility and abnomal gait from their 4 to 7 years (Fig. 2). These symtoms increased with age. When they came to our clinic, they were in early adolescence, and with short heights which was at least lower than -2 SD (<-2SD) compared with the average height of healthy Chinese adolescents at the same age (Table 1). Other joints, including knees, elbows, ankles and interphalangeal joints, showed no abnormalities. The hearing, vision, and intellectual development were normal in probands of Family 1 and 2, but in Family 3 and 4, the probands had myopia. X-rays of pelvis, both lower extremities and spines showed flattening of the acetabular roof, small capital femoral epiphyses or partial collapse of bilateral femoral heads, shortening of the femoral neck and base of the ilium, enlarged distal femur metaphyses, coxa vara, scoliosis and platyspondyly (Fig. 3). The parents had no similar signs or symtoms in Family 1, 2 and 3. In Family 4, the father (Ⅰ-1) and brother (Ⅱ-1) of the proband were normal, but the 46-year old mother of the proband (Ⅰ-2) was 141.4-cm tall (<-3SD). Pain in bilateral hip and knee joints appeared at her age of 20, and she had myopia and cataract. X-rays demonstrated rough articular surface, narrowing joint spaces, sclerotic edges of both acetabulums, concave on the upper and lower margin of multiple vertebral bodies.

#### Osteoarthritis with mild chondrodysplasia (OSCDP)

**Figure 4:**
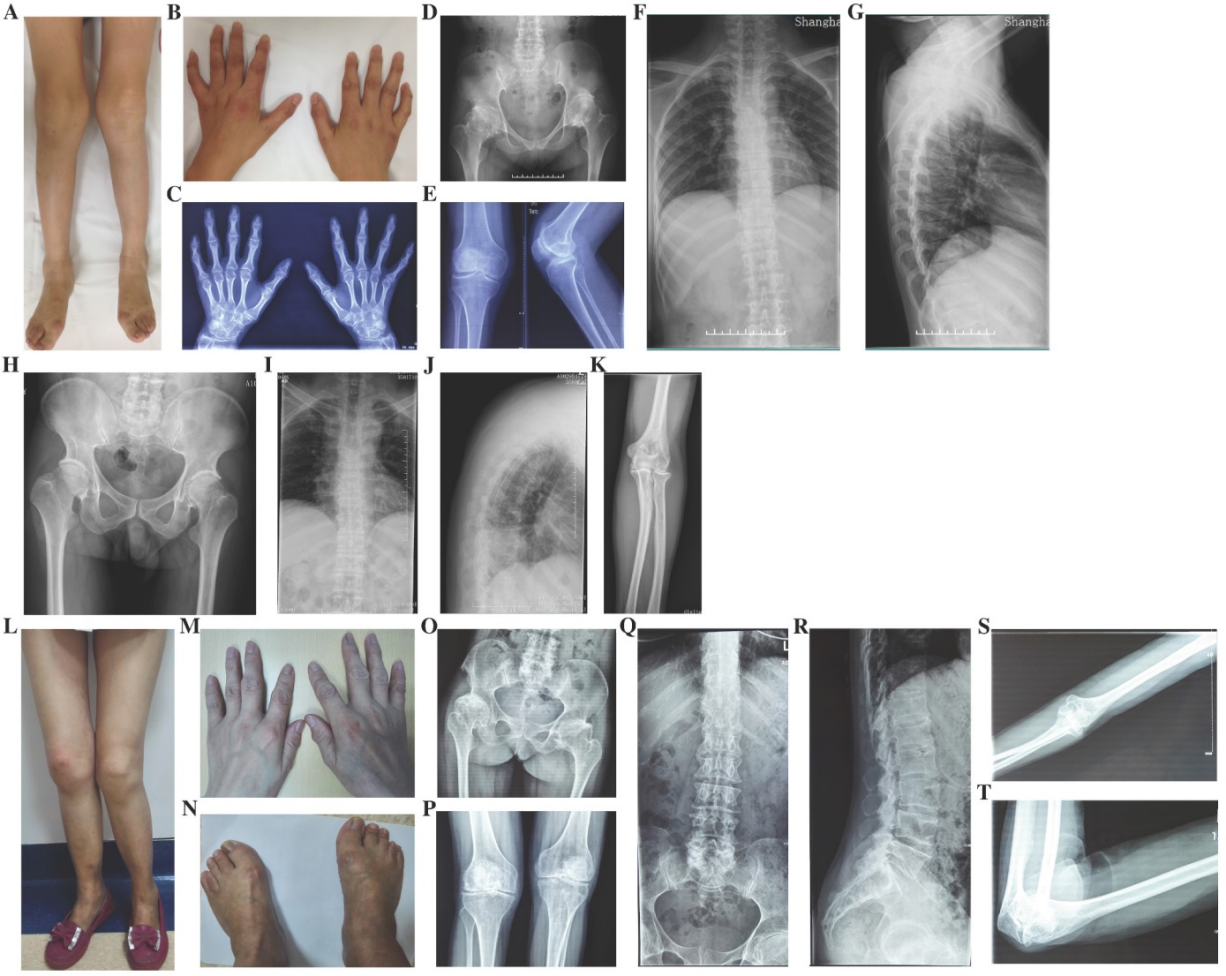
Photographs and radiographs of the probands with OSCPD and Czech dysplasia. A and B: Photographs of lower extremities (A) and hands (B) of the proband in Family 5 with OSCPD. Bilateral knees, ankles, multiple metacarpophalangeal and interphalangeal joints were enlarged. C-G: Radiographs of hands (C), pelvis (D), knees (E) and spine (F and G) of the proband in Family 5 with OSCPD. Expansion of metaphyses and stenosis of joint spaces were displayed in hands, osteoarthritis and dysplasia were shown in pelvis, and mild scoliosis, platyspondyly and narrowing spaces were obvious in spine and knees. H-K: Radiographs of pelvis (H), spine (I and J) and elbow (K) of the proband in Family 6 with OSCPD. Osteoarthritis were significant in pelvis, narrow intervertebral space and biconcave vertebaes were visible in spine. Osteoarthritis and osteophytes were observed in elbows. L-N: Photographs of lower extremities (L), hands (M) and feet (N) of the proband in Family 8 with Czech dysplasia. Bilateral knees, ankles, multiple metacarpophalangeal and interphalangeal joints were enlarged, and the fourth and fifth toes of both feet were shorter. O-T: Radiographs of pelvis (O), knees (P), spine (Q and R) and elbow (S and T) of the proband in Family 8 with Czech dysplasia. Obliquity, osteoarthritis and dysplasia were obvious in pelvis. Slight platyspondyly and narrow intervertebral spaces were significant in spine. Bony hyperplasia and narrow joint space were visible in elbows.

Three families including 19 affected individuals were diagnosed with OSCDP (Fig. 2.). They all had enlarged bilateral knees, elbows, wrists, ankles, multiple metacarpophalangeal and interphalangeal joints from a young age of 17 (16-18). Pain and motion limitation were also occured in above joints with time (Table 1). In Family 5 and 6, the affected members no matter males or females had short stature and the heights were at least lower than -1 SD (<-1SD), compared with the average height of healthy Chinese adults at the same age. Additionally, in Family 5, the 4 year-old niece (Ⅲ-1) of the proband was 85-cm tall (<-3SD) and disturbed by delayed growth. However, in Family 7, the male patients and female patients both had the normal height of 171.3cm (170.8-173.4cm) and 162.3cm (161.7-163.3cm), respectively. The hearing, vision, and intellectual development were normal in all 19 affected individuals. X-rays of pelvis showed visible osteoarthritis of bilateral hips including irregular shapes of the femoral heads, shortening femoral necks, the sclerosis of acetabular edges, and the narrow joint space, while radiographs of the spine revealed hyperosteogeny in edges of vertebral bodies, platyspondyly and narrow intervertebral spaces. Additionally, expansion of metaphyses and stenosis of joint spaces were observed in X-rays of the hands and knees. Osteoarthritis, osteophytes and narrowing space were observed in the X-rays of elbows (Fig. 4). 13 unaffected family members including Ⅲ-2 of Family 5, Ⅲ-3, Ⅳ-1, Ⅳ-2, Ⅳ-3 of Family 6, and Ⅲ-6, Ⅲ-15, Ⅳ-1, Ⅳ-4, Ⅳ-8, Ⅳ-9, Ⅳ-10, and Ⅳ-11 of Family 7, had no significant symptoms.

#### Czech dysplasia

In Family 8, the 45 year-old female proband (Ⅰ-2) was 146.3-cm tall (<-2SD) (Fig. 2). Since her age of 12, the enlargement and motion limitation of multiple interphalangeal joints, knees, ankles and elbows appeared successively. Additionally, the fourth and fifth toes of both feet were shorter. X-rays of pelvis showed obliquity, osteoarthritis and narrow joint space of bilateral hips, sclerosis of acetabulums, shortening femoral necks and destruction of articular cartilage. Radiographs of spine demonstrated slight platyspondyly and narrow intervertebral spaces. X-rays of other affected joints showed bony hyperplasia and narrow joint space (Fig. 4). In the family, the 24 year-old daughter of the proband (Ⅱ-1) was 151.4-cm tall (<-1SD), and had the similar symptoms, including shorter fourth and fifth toes since 14 years old (Table 1).

#### Epiphyseal dysplasia, multiple, with myopia and deafness (EDMMD)

**Figure 5:**
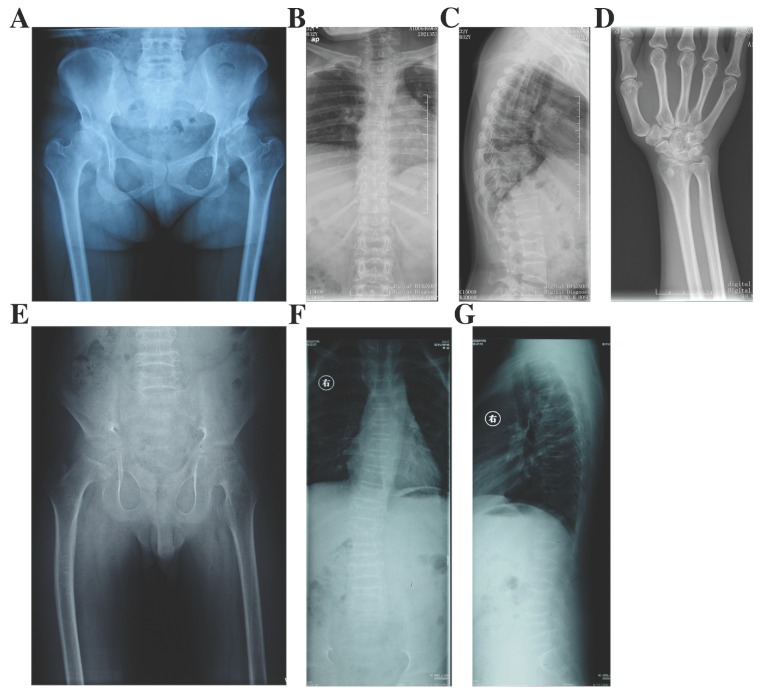
Radiographs of the probands with EDMMD and Kniest dysplasia. A-D: X-rays of pelvis (A), spine (B and C) and hands (D) of the proband in Family 9 with EDMMD. Osteoarthritis, coxa vara, shortening femoral necks and sclerosis of acetabulums were obvious in pelvis. Biconcave deformity, rough edges of vertebral body and narrow intervertebral disc spaces were significant in spine. Narrowing of the metacarpophalangeal and interphalangeal joint spaces and expansion of metaphyses were showed in hands and long tubular bones. E-G: Radiographs of pelvis (E) and spine (F and G) of the proband in Family 10 with Kniest dysplasia. Irregular shapes of femur heads, narrow joint space of bilateral hips, flat acetabular roof, coxa vara, severe multiple platyspondyly and slight scoliosis were showed.

In Family 9, the 32 year-old female proband (Ⅱ-1) had the height of 135 cm (<-3SD) (Fig. 2). Her growth was delayed since birth. She came to our department for pain in lumbar vertebra, and the swelling in both lower extremities from 15 years old. Moreover, she had myopia and complained the hearing loss of the right ear (Table 1). X-rays of pelvis showed narrow joint space of bilateral hips, coxa vara, shortening femoral necks and sclerosis of acetabulums. Radiographs of spine showed multiple thoracolumbar vertebras with biconcave deformity, rough edges of vertebral body and narrow intervertebral disc spaces. X-rays of hands displayed narrowing of the metacarpophalangeal and interphalangeal joint spaces and expansion of metaphyses in hands and long tubular bones (Fig. 5). There were no additional affected family members in this family.

#### Kniest dysplasia

In Family 10, the male proband (Ⅱ-1) was 12 years old, and 130.5-cm tall (<-2SD) (Fig. 2). From the age of eight, the proband complained for the pain in hip joints and lumbar vertebra. No abnormalities were found in his hearing, vision, and mental development (Table 1). X-rays demonstrated irregular shapes of femur heads, narrow joint space of bilateral hips, flat acetabular roof, coxa vara, severe multiple platyspondyly and slight scoliosis (Fig. 5). The father of the proband (Ⅰ-1) was 35 years old and 141.3-cm tall (<-3SD) but the mother of the proband (Ⅰ-2) was normal.

### Genetic analysis

**Figure 6:**
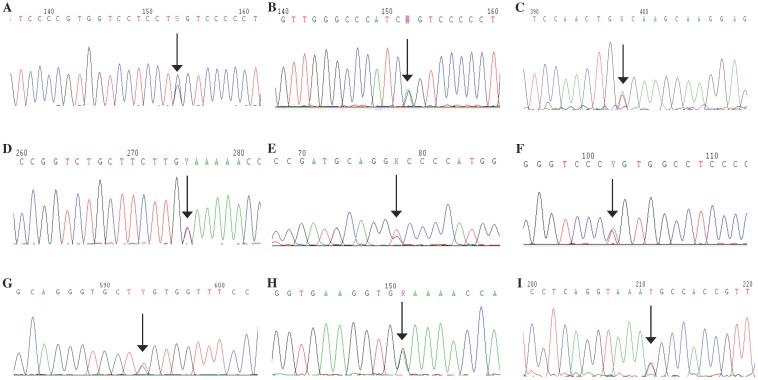
Mutation analysis. A: A missense mutation, p.Gly246Arg, in exon 11 of *COL2A1* was detected in the proband of Family 1. B: A missense mutation, a missense mutation in exon 23, p.Gly504Ser, in exon 23 was identified in the proband of Family 2. C: A missense mutation, Gly1014Val, in exon 45 was detected in the proband of Family 3. D: A missense mutation, p.Ter1488Gln, in exon 54 was identified in the proband and his mother of Family 4. E: A missense mutation, p.Gly204Val, in exon 9 was present in the proband, her father, sister and niece of Family 5. F: A missense mutation, p.Arg719Cys, in exon 33 was identified in two probands and 13 affected family members of Family 6 and Family 7. G: A missense mutation, p.Arg275Cys, in exon 13 was identified in the proband and her daughter of Family 8. H: A missense mutation, p.Gly678Glu, in exon 31 was found in the proband of Family 9. I: A splice site mutation, c.816+6C>T, in intron 12 was identified in the proband and his father of Family 10.

**Figure 7:**
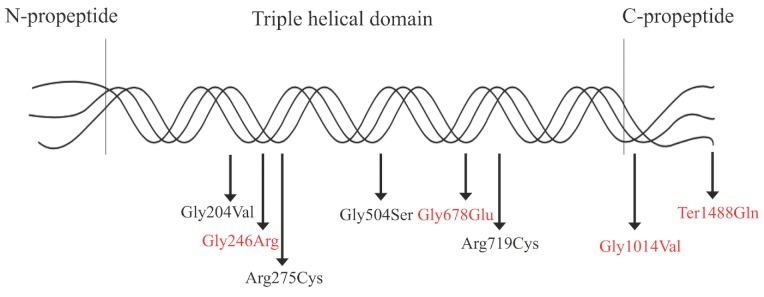
Schematic illustration of the type Ⅱ collagen trimer with disease-causing mutations related to *COL2A1*-related dysplasias. Mutations in red indicate novel mutations, and mutations in black indicate reported mutations.

In total, nine mutations were identified in the COL2A1 gene in the ten Chinese families, including four *de novo* mutations (Fig. 6). In Family 1, a missense mutation in exon 11, p.Gly246Arg (c.736G>C) was detected in the proband. In Family 2, only the proband harbored a missense mutation in exon 23, p.Gly504Ser (c.1510G>A). In Family 3, a missense mutation in exon 45, Gly1014Val (c.3041G>T) was detected in the proband. In Family 4, a missense mutation in exon 54, p.Ter1488Gln (c.4462T>C) was identified in the proband and his mother. In Family 5, the missense mutation in exon 9, p.Gly204Val (c.611G>T) was present in the proband, her father, sister, and niece. In Family 6 and 7, a missense mutation in exon 33, p.Arg719Cys (c.2155C>T) was identified in the probands and 13 affected family members. In Family 8, the proband and her daughter had a missense mutation in exon 13, p.Arg275Cys (c.823C>T). In Family 9, a missense mutation in exon 31, p.Gly678Glu (c.2033G>A) was found in the proband. In Family 10, a splice site mutation in intron 12, c.816+6C>T, was identified in the proband and his father. The above mutations we identified in COL2A1 gene were not detected in the 24 unaffected family members or the 250 healthy volunteers. Of all the mutations, five (c.816+6C>T, p.Gly246Arg, p.Gly678Glu, p.Gly1014Val and p.Ter1488Gln) were novel and four (p.Gly204Val, p.Arg275Cys, p.Gly504Ser and p.Arg719Cys) were reported previously, based on HGMD and all missense mutations were predicted as disease-causing through Polyphen-2, SIFT and MutationTaster (Fig. 7).

### Prenatal diagnosis and genetic counseling

At the age of 26, the proband in Family 5 with OSCDP had completed prenatal diagnosis and genetic counseling for her second fetus in our department through amniocentesis. At 20th week of gestation, amniocentesis was guided by ultrasound and 20 mL of amniotic fluid was obtained. DNA of the fetus was extracted with TGuide cells/tissue genomic DNA kit [TIANGEN BIOTECH (BEIJING) CO., LTD, China]. After amplifying all exons and exon-intron boundaries of COL2A1 gene with PCR, Sanger sequencing was performed aiming at the same mutation site identified in the mother. As a result, the fetus also carried the missense mutation in exon 9, p.Gly204Val (c.611G>T), indicating the fetus might have same symptoms with the mother in the future.

## Discussion

In the present study, ten Chinese families were classified into SEDC, OSCDP, Czech dysplasia, EDMMD and Kniest dysplasia, based on clinical characteristics and genetic screening. To the best of our knowledge, OSCDP, Czech dysplasia and EDMMD in Chinese were reported herein for the first time. Nine mutations were identified including five novel and four reported previously. Moreover, prenatal diagnosis and genetic counseling were accomplished in the female proband with OSCDP in Family 5 through amniocentesis. These findings would be useful to determine clinical and molecular phenotypes and make up for *COL2A1*-related disorders in Chinese populations.

The phenotypic spectrum of *COL2A1*-related disorders is very wide. SEDC is characterized with disproportionate short stature especially short trunk, abnormal epiphyses, flattened vertebral bodies and hypoplastic odontoid. Skeletal features are manifested at birth and developed with time. Other features include myopia, retinal degeneration with retinal detachment and cleft palate 18-20. The radiological features of SEDC are retarded ossifications of vertebral bodies, pelvis and lower extremities 18. OSCDP and Czech dysplasia share similar clinical manifestations of early-onset and progressive pseudorheumatoid arthritis due to defects in cartilage. Athralgia, swelling and stiffness occur in multiple joints including metacarpophalangeal joints, interphalangeal joints, shoulders, elbows, hips and knees successively 21-23. In spite of similarities, the distinctive features of OSCDP are spinal deformity and hypoplastic pelvis, while the characteristic manifestations of Czech dysplasia are platyspondyly, short third and fourth toes, and sensorineural hearing loss 24. Like X-ray features of primary osteoarthritis, typical radiographic findings of OSCDP and Czech dysplasia are narrow joint space, subchondral sclerosis and ostreophytes at joint margins 25. In EDMMD, short stature, brachydactyly and genu valgus deformity are common, whereas early-onset progressive myopia, retinal thinning, asteroid hyalosis or crenated cataracts may accompany 26. Radiographic studies show general dysplasia of epiphyses and concave vertebral bodies 26. In Kniest dysplasia, the classical phenotype is short trunk dwarfism due to severely affected skeletal growth, scoliosis, platyspondyly and arthropathy, while extraskeletal features mainly include myopia, prominent eyes, conductive hearing loss and mid-face hypoplasia 3. The X-ray findings of Kniest dysplasia display dumbbell-shaped long bones, platyspondyly, and enlarged joints 27.

As a result of the different mutation types and mutation locations, the genotypic spectrum of *COL2A1*-related disorders also varies widely. In the present study, three substitutions of glycine with bulkier amino acids in Gly-X-Y repeats (p.Gly246Arg, p.Gly504Ser and p.Gly1014Val) and one substitution of stop codon with glutamine (p.Ter1488Gln) were identified with SEDC. The substitutions lead to dramatic impairment in protein assembly and stability 28. Patients with C-propetide glycine substitutions have been reported to be shorter than those with N-propetide substitutions 17. Patients with p.Gly1014Val or p.Ter1488Gln have myopia or cataract, which may indicate that mutations in C-propetide are tend to result in ocular changes. Among the three glycine substitutions associated with SEDC, the glycine to serine substitution caused milder phenotypes in Family 2, while glycine to nonserine substitutions produce more severe phenotypes including severe scoliosis, significantly hypoplastic pelvis or coxa vara in Family 1 and Family 3. In addition, p.Gly204Val, p.Arg650Cys and p.Arg719Cys were detected in patients with OSCDP, and p.Arg275Cys was detected in patients with Czech dysplasia. In Family 6 and 7, the same mutation caused similar manifestations, however difference in height was obvious. This may suggest phenotypic heterogeneity. Compared with the glycine to valine substitution at position 1014, the same substitution at position 204 was mainly involved in joints and resulted in milder phenotypes in spine and pelvis. The results confirmed that mutations in C-propetide region caused severe phenotypes. Affecting the supramolecular organization of collagen fibrils and the transport and secretion of molecules, arginine-to-cysteine substitutions always cause moderate phenotypes with precocious osteoarthritis but never a severe and perinatally lethal condition 9, 29. Substitution of arginine residue in the X position of Gly-X-Y motifs results in disorders with ocular involvement while substitutions in the Y position cause no ocular anomalies 9. The mutation p.Arg275Cys always results in deformity in toes and is regarded as a mutation hot spot of *COL2A1*-associated Czech dysplasia 7. In the female patient with EDMMD, p.Gly678Glu was identified, while in a case with multiple epiphyseal dysplasia (MED), a similar mutation, p.Gly678Arg has been reported 30. The patients with MED had not myopia or hearing loss. Since the amino acid glutamine is much larger than arginine and has low pH, the different amino acid mutations at the same position may result clinical phenotypes of different severities. In patients with Kniest dysplasia, a novel splice-site mutation, c.816+6C>T was detected, and splice-site mutations always lead to exon skipping and cause STL1 or Kniest dysplasia.

## Conclusion

In the present study, ten Chinese families with *COL2A1* mutations were classified into five categories of dysplasia, including SEDC, OSCDP, Czech dysplasia, Kniest dysplasia and EDMMD. Among them, OSCDP, Czech dysplasia and EDMMD were first reported in Chinese patients, with five novel *COL2A1* mutations being discovered. Our results further demonstrated that phenotypic and genotypic spectra of *COL2A1*-related disorders are very wide. The new findings reported herein would contribute to the investigations of the phenotype-genotype relations among patients with *COL2A1*-related dysplasia.

## Figures and Tables

**Table 1:** Clinical data and mutations identified in *COL2A1* from patients with *COL21A*-related dysplasias.

Family	Patient	Diagnosis	Gender	Age (y)	Height (cm)	Onset age (y)	Platyspondyly	Osteoarthritis	Toes deformity	Myopia	Hearing loss	DNA change	Protein change	Novel
F1	Ⅱ-1	SEDC	M^a^	5	100.0	4	+^c^	-^d^	-	-	-	c.736G>C	p.Gly246Arg	Yes
F2	Ⅱ-1	SEDC	M	13	116.5	5	+	-	-	-	-	c.1510G>A	p.Gly504Ser	No
F3	Ⅱ-1	SEDC	M	12	128.1	7	+	-	-	+	-	c.3041G>T	p.Gly1014Val	Yes
F4	Ⅱ-2	SEDC	M	10	120.8	4	+	-	-	+	-	c.4462T>C	p.Ter1488Gln	Yes
F4	Ⅰ-2	SEDC	F^b^	46	141.4	20	+	+	-	+	-	c.4462T>C	p.Ter1488Gln	Yes
F5	Ⅱ-4	OSCDP	F	25	151.4	8	+	+	-	-	-	c.611G>T	p.Gly204Val	No
F5	Ⅱ-2	OSCDP	F	27	134.5	10	+	+	-	-	-	c.611G>T	p.Gly204Val	No
F5	Ⅰ-1	OSCDP	M	54	145.0	16	/^e^	/	-	-	-	c.611G>T	p.Gly204Val	No
F5	Ⅲ-1	OSCDP	F	4	85.0	Not yet	/	/	-	-	-	c.611G>T	p.Gly204Val	No
F6	Ⅲ-1	OSCDP	M	40	154.0	16	+	+	-	-	-	c.2155C>T	p.Arg719Cys	No
F6	Ⅲ-5	OSCDP	M	26	167.4	14	+	+	-	-	-	c.2155C>T	p.Arg719Cys	No
F6	Ⅲ-6	OSCDP	M	38	154.3	16	+	+	-	-	-	c.2155C>T	p.Arg719Cys	No
F6	Ⅲ-7	OSCDP	F	35	148.6	16	+	+	-	-	-	c.2155C>T	p.Arg719Cys	No
F6	Ⅱ-5	OSCDP	M	65	153.0	20	/	/	-	-	-	c.2155C>T	p.Arg719Cys	No
F6	Ⅱ-6	OSCDP	F	62	146.0	20	/	/	-	-	-	c.2155C>T	p.Arg719Cys	No
F7	Ⅲ-7	OSCDP	M	45	173.4	18	+	+	-	-	-	c.2155C>T	p.Arg719Cys	No
F7	Ⅲ-1	OSCDP	M	49	170.8	16	/	/	-	-	-	c.2155C>T	p.Arg719Cys	No
F7	Ⅲ-4	OSCDP	F	47	162.5	18	/	/	-	-	-	c.2155C>T	p.Arg719Cys	No
F7	Ⅲ-9	OSCDP	M	44	171.3	18	+	+	-	-	-	c.2155C>T	p.Arg719Cys	No
F7	Ⅲ-14	OSCDP	F	46	165.7	19	/	/	-	-	-	c.2155C>T	p.Arg719Cys	No
F7	Ⅱ-2	OSCDP	M	71	168.9	16	/	/	-	-	-	c.2155C>T	p.Arg719Cys	No
F7	Ⅱ-6	OSCDP	F	68	160.6	17	/	/	-	-	-	c.2155C>T	p.Arg719Cys	No
F7	Ⅳ-2	OSCDP	M	27	177.3	17	/	/	-	-	-	c.2155C>T	p.Arg719Cys	No
F7	Ⅳ-3	OSCDP	F	24	162.1	18	/	/	-	-	-	c.2155C>T	p.Arg719Cys	No
F8	Ⅰ-2	Czech dysplasia	F	45	146.3	12	+	+	+	-	-	c.823C>T	p.Arg275Cys	No
F8	Ⅱ-1	Czech dysplasia	F	24	151.4	14	+	+	+	-	-	c.823C>T	p.Arg275Cys	No
F9	Ⅱ-1	EDMMD	F	32	135.0	15	+	+	-	+	+	c.2033G>A	p.Gly678Glu	Yes
F10	Ⅱ-1	Kniest dysplasia	M	12	130.5	8	+	+	-	-	-	c.816+6C>T	Unknown	Yes
F10	Ⅰ-2	Kniest dysplasia	M	35	141.3	13	/	/	-	-	-	c.816+6C>T	Unknown	Yes

^a^M, male; ^b^F, female; ^c^ “+” indicates that the patient has the symptom; ^d^ “-” indicates that the patient does not have the symptoms; ^e^ “/” indicates that it is unclear whether the patient has the symptoms or not.

## Abbreviations

SEDC: spondyloepiphyseal dysplasia congenita
OSCPD: osteoarthritis with mild chondrodysplasia
EDMMD: epiphyseal dysplasia, multiple, with myopia and deafness
ACG2: achondrogenesis type 2
HCG: hypochondrogenesis
PLSDT: platyspondylic dysplasia, Torrance type
SED Strudwick type: spondyloepiphyseal dysplasia, Strudwick type
SPPD: spondylopepripherad dysplasia
STL1: Stickler syndrome type 1
LCPD: Legg-Calve-Perthes disease
PCR: polymerase chain reaction
MED: multiple epiphyseal dysplasia
SNPs: single nucleotide polymorphisms
